# Chest wall reconstruction with implantable cross-linked porcine dermal collagen matrix: Evaluation of clinical outcomes

**DOI:** 10.1016/j.xjtc.2022.01.021

**Published:** 2022-02-22

**Authors:** Alessandro Gonfiotti, Domenico Viggiano, Eduart Vokrri, Marco Lucchi, Duilio Divisi, Roberto Crisci, Felice Mucilli, Federico Venuta, Luca Voltolini

**Affiliations:** a Section of Thoracic Surgery, Department of Cardio-Thoracic and Vascular Surgery, Azienda Ospedaliero Universitaria Careggi, Firenze, Italy; b Section of Thoracic Surgery, Department of Cardio-Thoracic and Vascular, Azienda Ospedaliero Universitaria Pisana, Pisa, Italy; c Thoracic Surgery Unit, Ospedale Civile Giuseppe Mazzini, Università degli Studi L'Aquila, Teramo, Italy; d General and Thoracic Surgery, Department of Medical Science, Università degli Studi G. d'Annunzio, Chieti-Pescara, Italy; e Thoracic Surgery Unit, Azienda Ospedaliero Universitaria Policlinico Umberto I, Roma, Italy

**Keywords:** chest wall reconstruction, chest wall tumor, biological mesh, titanium bar, cross-linked porcine dermal collagen matrix, CWR, chest wall reconstruction, LD, latissimus dorsi, PACLIDEM, porcine-derived acellular cross-linked dermal matrix, PCWT, primary chest wall tumor, PM, pectoral major, PMCWT, primary malignant chest wall tumor, PTFE, polytetrafluoroethylene, SCWT, secondary chest wall tumor

## Abstract

**Objectives:**

The aim of the study is to evaluate clinical applications, safety, and effectiveness of a porcine-derived acellular cross-linked dermal matrix biological mesh in chest wall reconstruction.

**Methods:**

We retrospectively analyzed a prospective multicenter database of chest wall reconstructions using a biological mesh in adult patients undergoing operation between October 2013 and December 2020. We evaluated preoperative data, type of resection and reconstruction, hospitalization, 30-day morbidity and mortality, and overall survival.

**Results:**

A total of 105 patients (36 women [34.2%]; mean age, 57.0 ± 16.1 years; range, 18-90 years) were included, they have admitted for: primary chest wall tumor (n = 52; 49.5%), secondary chest wall tumor (n = 29; 27.6%), lung hernia (n = 12; 11.4%), trauma (n = 10; 9.6%), and infections (n = 2; 1.9%). The surgical sites were preoperatively defined as at high risk of infection in 28 patients (26.7%) or as infected in 16 (15.2%) patients. Thirty-days morbidity was 30.5% (n = 32 patients); 14 patients (13.3%) had postoperative complications directly related to chest wall surgical resection and/or reconstruction. We experienced no 30-day mortality; 1-year and 2-year mortality was 8.4% and 16.8%, respectively.

**Conclusions:**

Biological mesh represents a valuable option in chest wall reconstruction even when surgical sites are infected or at high-risk of infections. This mesh shows low early and late postoperative complication rates and excellent long-term stability.

**Figure:**
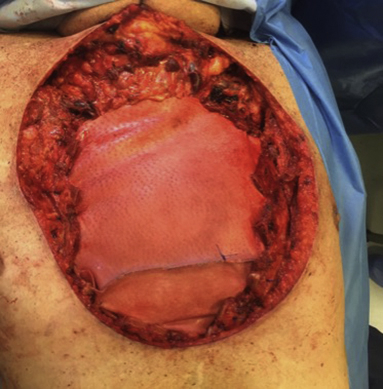
Anterior chest wall reconstruction with cross-linked porcine dermal acelluar collagen mesh.

**Central Message**

Biological mesh represents a valuable option in chest wall reconstruction, especially in infected or high-risk-of-infection surgical sites.

**Perspective**

Chest wall reconstruction is a challenge for surgeons. We report a large, multicenter experience in using porcine-derived acellular cross-linked dermal matrix for chest wall reconstruction. Biological mesh showed excellent results in terms of stability, wound healing, and no complications have been reported, suggesting that this material is a safe and valuable option in chest wall reconstruction.

See Commentary on page 261.

Despite the wide range of prosthetic materials available, the ideal method for chest wall reconstruction (CWR) is still a matter of debate, even if this surgical procedure clearly improves postoperative ventilation, shortens overall hospital stay, and aids postoperative pulmonary physiology and mechanics.^1^, ^2^, ^3^, ^4^, ^5^ The primary challenge for surgeons is the difficulty in predicting the nature and extension of chest wall defect because the excision can result in partial- or full-thickness thoracic wall defects.^6^, ^7^, ^8^ Currently, there are 2 ways to cover chest wall defects: prosthetic or biologic mesh and/or soft tissue flaps.^9^, ^10^, ^11^, ^12^ The introduction of biological prosthetic materials represents an innovation in CWR. Initially used during the 1990s, extracellular biological meshes provide the extracellular scaffold necessary for tissue healing. Biological meshes can either derive from human (allograft; derived from dermis, intestinal mucosa, or pericardium) or animal (xenograft; usually porcine or bovine) tissues. Most published studies on biological meshes are case series, and their use is limited to contaminated or infected fields for abdominal wall reconstruction where a synthetic mesh is considered strongly contraindicated.^7^, ^8^, ^9^

Here, we show a large, multicentric retrospective series reporting the use of the porcine-derived acellular cross-linked dermal matrix (PACLIDEM) for CWR, either alone or in combination with rigid reconstruction. The purpose is to confirm clinical applications, to assess safety and performance, and to evaluate short-term and long-term patient outcomes following the use of biological meshes in CWR.

## Materials and Methods

Retrospectively, we analyzed a prospective multicenter database of CWR using the PACLIDEM mesh 1.5-mm thick named Permacol (Covidien) in the following Italian university hospitals: Firenze, Pisa, Roma-Umberto I, L'Aquila-Teramo, and Chieti.

We evaluated the data in the database from the first case during October 2013 through December 31, 2020. The use of the biological tissue matrix was approved by the clinical directorates of the involved institutes. Indications for using biological mesh and including patients in the database were: CWR in oncology patients, CWR after trauma, CWR in lung hernia, and CWR after resections for infectious disease.

When its potential use was considered in the preoperative planning, patients were advised, and informed consents were obtained; all patients provided also an informed consent for the publication of their study data. Patients younger than age 18 years were excluded. Muslim patients were warned of the porcine origin and biochemical characteristics, including the decellularization of the dermis and therefore the absence of genetic material (ie, DNA). The study was approved first by the University Hospital Careggi (Florence) Institutional Review Board (ID: 09, April 2020) and then by the other involved institutes.

### Surgical Policies Followed in the Series

#### Primary or secondary tumors

Our surgical policy in the treatment of chest wall tumors was, in general: skin incision included the site of the previous biopsy (in case present) and the invaded skin or previously irradiated tissues; wide resection of a lateral tumor included the affected ribs with at least 3-cm free margin proximally and distally to the tumor and the adjacent portions of one normal rib above and below the lesion; the extent of surgery in sternal primary tumors (partial subtotal or complete sternectomy) depended on the dimension and the location of the tumor and, in all cases, resection included the adjacent sternocostal cartilages on each side; tumor extension into the chest cavity was evaluated and any other structure involved in the tumor was also excised. Resection and reconstruction were performed as a 1-stage procedure in all cases. Every effort was made to wean patients rapidly from the ventilator. The need to perform induction chemotherapy or postoperative chemotherapy and radiotherapy in the case of high-grade sarcomas was discussed and planned with the medical oncologist and the radiotherapist in an oncological multidisciplinary group.

#### Trauma, lung hernia, and infections

In case of benign disease, the reconstruction first aimed to restore chest wall stability, removing, in case of trauma, bone fragments from the pleural cavity and reducing rib and sternal fractures as much as possible. In case of infection, the resection was limited to the compromised bones.

Lung herniation usually results from the loss of the intercostal muscle (sometimes of 2 adjacent intercostal spaces); therefore, primary closure cannot be an option. Indication to surgical correction of lung hernia was the presence of parenchymal herniation, with paradoxical movement of the lung outside the chest, which led lung trauma documented by computed tomography scan and/or by symptoms like hemoptysis or recurrent pneumonia.^13^ Even if direct closure has been previously described, based on the anatomy of the defect and the available tissues, we felt more confident using a mesh repair.^14^ The clinical and radiological absence of lung herniation during the follow-up period was considered as measure of success.

#### Drains

Pleural drains were placed whenever the pleural cavity was opened during surgery; policy for their removal was no air leak in the past 24 hours and fluid production < 3 mL/kg/24 hours. As a rule, a Redon soft tissue drain was always positioned, mainly in case of an associated muscle flap associated; policy for their removal was liquid production of serous quality < 50 mL/24 hours.

### Data Collected in the Clinical Database

We evaluated preoperative data such as demographic characteristics, gender, comorbidities, body mass index, chest wall disease, and indication for surgery. Type of resection and reconstruction were considered, including the use of rigid reconstruction or not and soft tissue coverage with muscular or muscular-cutaneous flap. We also considered the postoperative results evaluating short- and midterm complications (including postsurgery complications and prosthesis complications), the presence of paradoxical respiration movements clinically and/or radiologically observed (ie, lung hernia), hospitalization time, and 30-days and overall survival. Complications were defined as any deviation from the standard postoperative course^15^ and recorded according to the Clavien-Dindo classification.^16^

**Figure 1:**
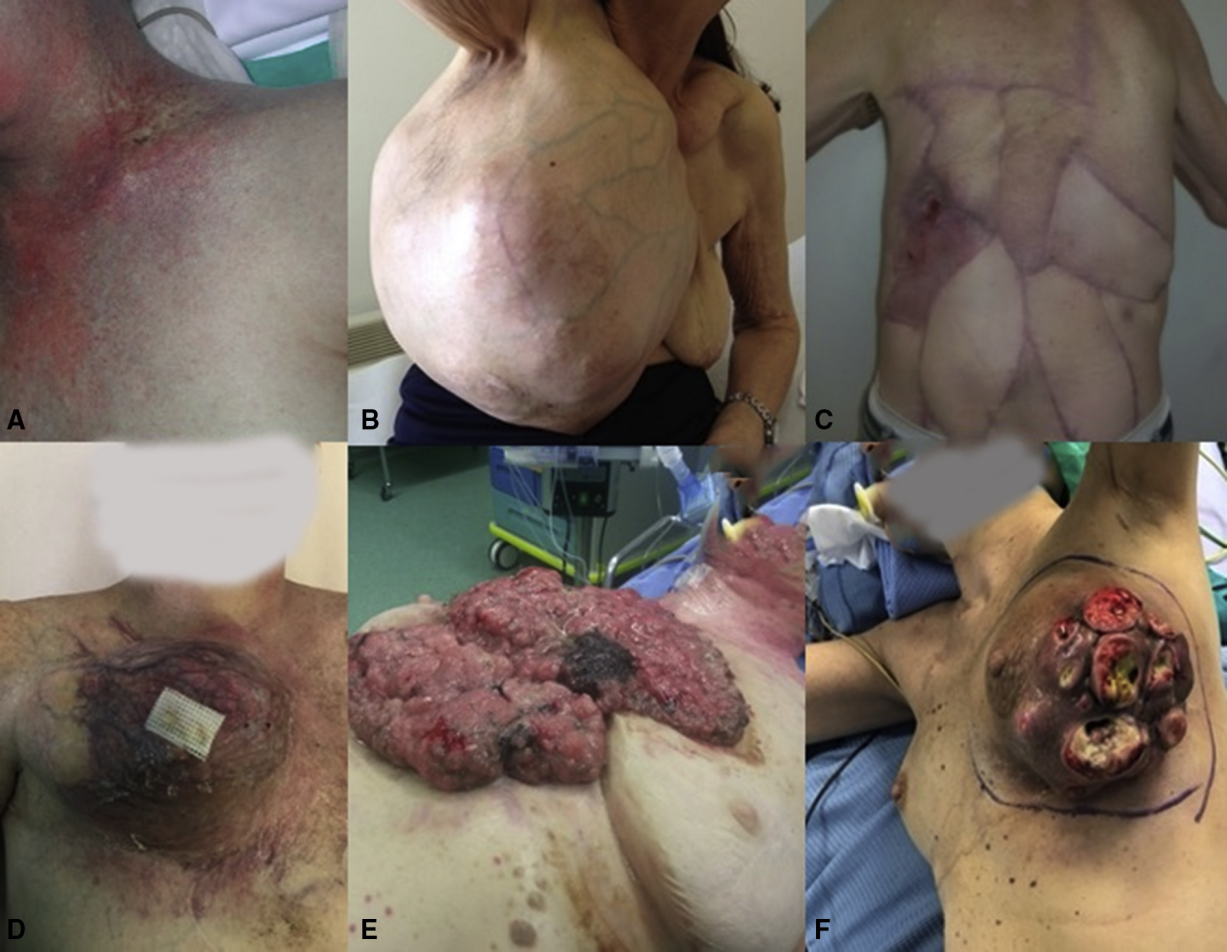
Surgical site classification. Sites with high risk of infection: A, Previous radiotherapy. B, Giant tumors in which a large amount of prosthetic material is planned for chest wall reconstruction. C, Redo chest wall surgery. Infected sites: D, Extensive soft tissue infiltration with ischemia. E, External cancer vegetation. F, Cancer ulceration.

Based on their preoperative characteristics (Figure 1), the surgical sites were classified as:

• Normal site
• High risk of postoperative infection; that is, the surgical site has undergone previous local treatments (eg, surgery or radiotherapy), cancer infiltration of soft tissues without skin involvement, and giant tumors in which a large amount of prosthetic material is planned for CWR
• Infected; that is, cancer with skin ulceration (ie, local relapse from breast cancer); or Trauma with loss of soft tissues and exposed lesions.

In case of oncological surgery, resection margins were classified following the Enneking classification,^17^ adapted to chest wall surgery, as follows: wide, marginal, or intralesional. The categories wide and marginal correspond to R0 resection. The Enneking category radical corresponds to limb amputation and cannot be used in CWR. In case of intralesional resection, the further subdivision in R1 (microscopic infiltration) and R2 (macroscopic infiltration) was used too.

Follow-up consisted in radiological examination (chest radiograph and/or computed tomography scan), outpatient visit, or telephone interview (depending on the disease and the interval from treatment). Data are presented with median and interquartile range for continuous variables and percentage for discreet variables.

## Results

Between October 2013 and December 2020, 105 patients (36 women [34.2%]; mean age, 57.0 ± 16.1 years; range, 18-90 years) had a porcine-derived acellular cross-linked dermal matrix implanted and were registered in the prospective database by the involved institutes. The indications for using biological mesh implant in chest wall repair/CWR were: primary chest wall tumor (PCWT) (n = 52 [49.5%]), secondary chest wall tumor (SCWT) (n = 29 [27.6%]), lung hernia (n = 12 [11.4%]), trauma (n = 10 [9.6%]), and infections (n = 2 [1.9%]). Forty-eight (45.7%) patients received the device alone, whereas in 60 (57.1%) patients, biological mesh was associated with titanium bars; 61 (58.1%) patients underwent also a myocutaneous flap and 26 (24.8%) patients underwent a simple muscular flap. Preoperative classification of surgical site was: infected (16 [15.2%]), high risk of infection (28 [26.7%]), and normal (61 [58.1%]). Chest wall defect was located as follows: anterior or anterolateral (52 [49.5%]), lateral (39 [37.1%]), and posterior (14 [13.4%]). R0 resection was achieved in 79 out of 81 tumor resections (97.5%).

Mean follow-up was 30.4 ± 20.1 months (range, 01-84). One patient (0.95%) was lost at follow-up after 22 months. Thirty-days morbidity was 30.5% (n = 32 patients): Clavien-Dindo grade I n = 17; grade II n = 12; grade III n = 2, and Grade IV n = 1.

The most represented adverse events were: complications anatomically related to the chest wall surgical resection and/or CWR site (14 [13.3%]), anemia requiring transfusion (8 [7.6%]), atrial fibrillation (5 [4.8%]), fever (2 [1.9%]), broncho-pleural fistula in a lobectomy (1 [0.9%]), and other (2 [1.9%]). The surgical complications (14 [13.3%]) were myocutaneous flap ischemia/necrosis (2 [1.9%]), bleeding with hemothorax (treated conservatively) (3 [2.9%]), wound hematoma or seroma (8 [7.6%]), and respiratory failure linked to impairment of chest wall movement (1 [0.9%]). In 1 case of lung hernia repair, the wound hematoma required a surgical revision so that the prosthesis was removed and then repositioned once the hemostasis was achieved.

**Figure 2:**
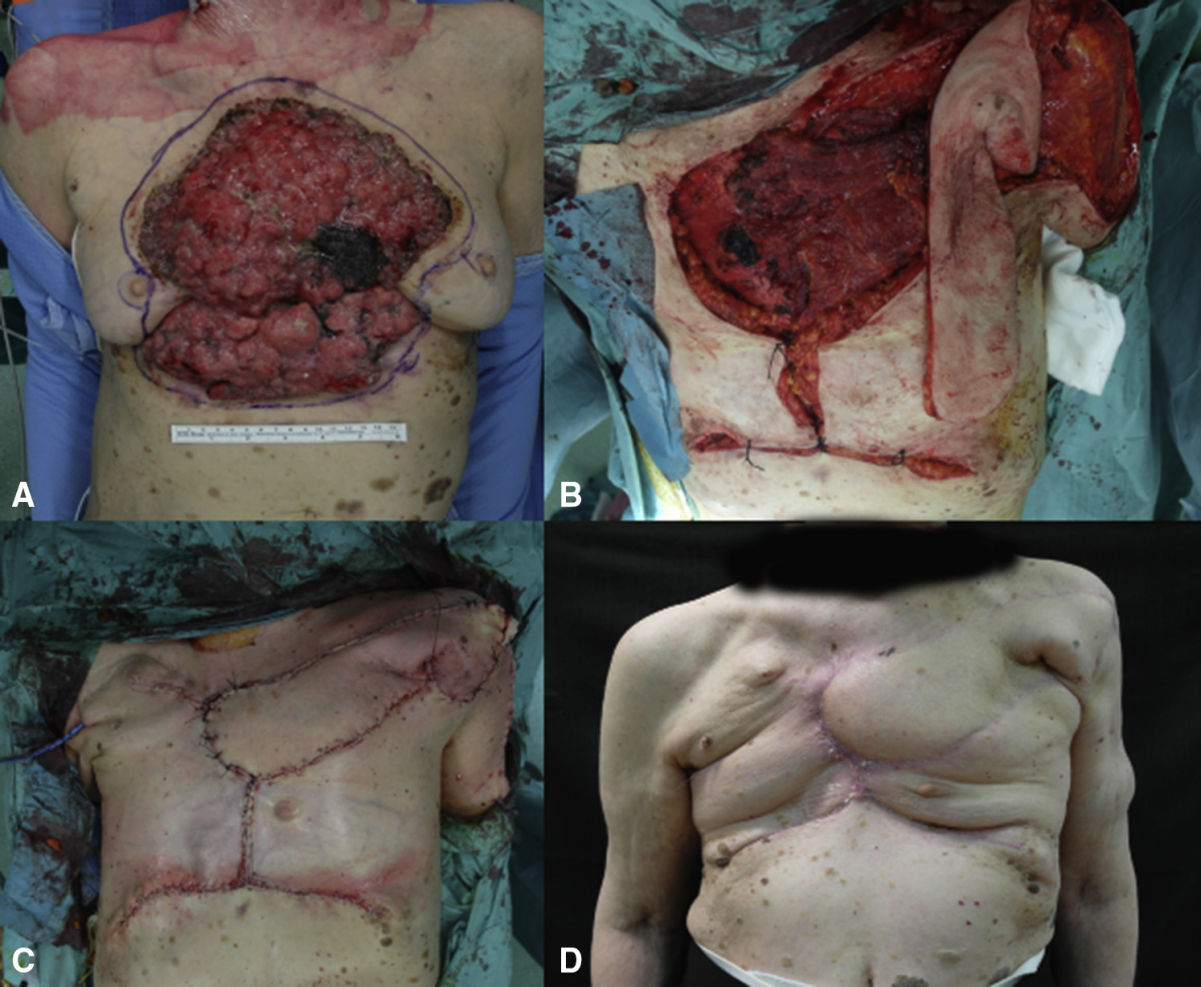
The case of a sternal tumor with a wide skin infiltration. A, Preoperative view. B, Muscular and cutaneous flap. C, Intraoperative result. D, Late cosmetic result.

**Figure 3:**
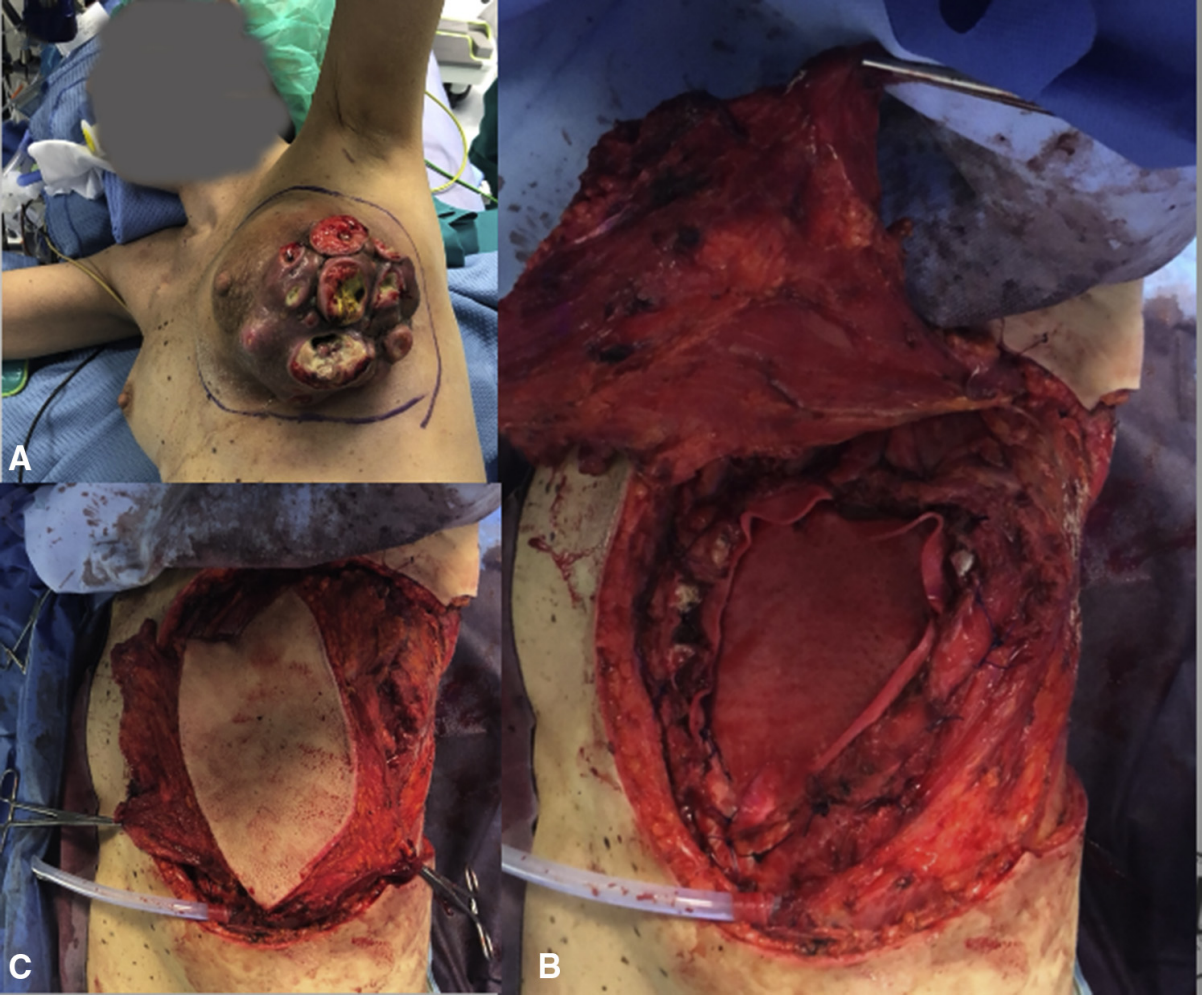
The case of a breast cancer recurrence infiltrating the skin. A, Preoperative view. B, Muscular and cutaneous omolateral flap. C, The flap covers the defect.

**Figure 4:**
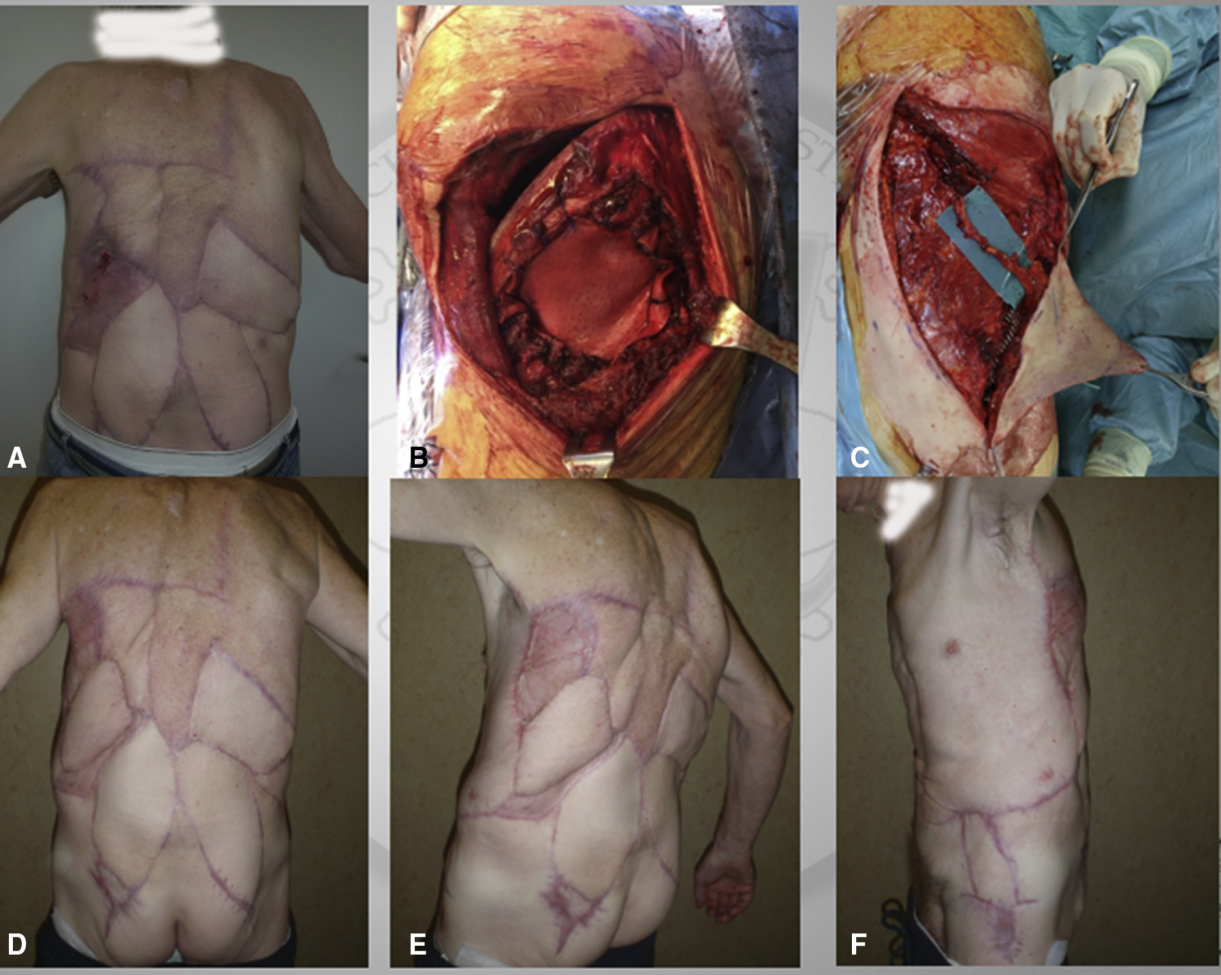
The case of a re-redo surgery for a local relapsed chest wall sarcoma. A, Preoperative view; the lesion is ulcerated on the skin. B, Chest wall resection and reconstruction with biological mesh. C, Muscolocutaneous perforator flap prepared. D-F, Final result.

Regarding the PACLIDEM implant, we experienced no prosthesis infection, no patient required prosthesis removal because of its detachment or rupture, and no patient had paradoxical respiration movement impairing respiratory function. No 30-day mortality was observed. The analysis of survival rates and curves depends on the underlying disease and this kind of study is beyond our intention and will not be evaluated (Figure 2, Figure 3, Figure 4 and Video 1) illustrate different cases of chest wall resection and reconstruction from this series. Due to the heterogeneity of the indications, the patients have been divided into 4 homogeneous groups and each group has been separately analyzed: group 1 = chest wall tumors, group 2 = trauma, group 3 = lung hernia, and group 4 = infectious disease.

### Group 1: Chest Wall Tumors

This is the largest group with 81 patients: PMCWTs n = 48 (59.2%); SCWTs n = 29 (35.8%), and benign lesions: n = 4 (4.9%). Main results are summarized in Table 1.

**Table 1:** Main characteristics of patients with chest wall tumors

Characteristic	Primary malignant chest wall tumors	Secondary chest wall tumors	Benign lesions
No. of participants	48 (59.2)	29 (35.8)	4 (4.9)
Age (y)	49.2 ± 15.9	66.8 ± 9.6	39.5 ± 3.0
Comorbidity	25 (52)	20 (70)	–
Resection			
Mean ribs removed∗	2.9	–	–
Total sternectomy	2 (4.2)	3	–
Partial sternectomy	16 (33)	6 (20.7)	–
Clavicular resection	1 (3.4)	1 (3.4)	–
Extended to other organs	–	21 (72)	–
Dimension of the biological mesh			
15 × 20 in	26 (54.2)	–	–
20 × 30 cm	6 (12.5)	12 (41.4)	–
10 × 15 cm	11 (22.9)	–	1 (25)
10 × 10 cm	1 (2.1)	–	2 (50)
10 × 5 cm	3 (6.3)	–	–
18 × 28 cm	1 (2.1)	–	–
15 × 20 cm	–	11 (37.9)	1 (25)
Smaller	–	6 (20)	–
Reconstruction: biological mesh with			
Rigid reconstruction	28 (56.3)	18 (62)	2 (50)
Muscular flap	37 (77.1)	29 (100)	0
Perioperative major complications	0	0	0
Mean hospitalization (d)	10.8 ± 9.9	13.5 ± 10.9	5.0 ± 1.1
30-d mortality	0	0	0
Overall survival	77 (33.9 ± 1.21)	37.9 (26.7 ± 20.65)	100 (52)

Values are presented as n (%), mean ± SD, or % (mean follow-up [mo] ± SD).

∗ Range, 1 to 8.

#### PMCWT

We performed 22 anterolateral (45.8%), 19 lateral (39.6%), and 7 posterior (14.6%) resections, with a mean number of 2.9 ribs removed (range, 1-8). Rigid reconstruction with titanium bars was necessary in n = 28 (56.3%) cases, and muscular flap was associated in 37 patients (77.1%): latissimus dorsi (LD) n = 15 (31.3%), pectoralis major (PM) n = 13 (27.1%), and other n = 9 (18.7%). Histology types are depicted in Table 2.

**Table 2:** Histopathology findings in primary and secondary malignant chest wall tumors

Histology type	n	%
Primary malignant chest wall tumors		
Chondrosarcoma	31	64.6
Grade I	21	67.7
Grade II	9	29.0
Grade III	1	3.2
Osteosarcoma	2	4.2
Ewing sarcoma	3	6.3
Radio-induced sarcoma	2	4.2
Solitary plasmacytoma	2	4.2
Desmoid tumor	2	4.2
Other sarcomas	6	12.5

Histology type	n	%
Secondary malignant chest wall tumors		
Non–small cell lung cancer	12	41.4
Mesothelioma	3	10.3
Breast cancer	7	24.1
Colonic cancer	1	3.4
Lymphoma	1	3.4
Renal cancer	2	6.9
Thyroid cancer	1	3.4
Hepatocellular carcinoma	1	3.4
Thymic carcinoma	1	3.4

Forty-six patients (96%) were extubated at the end of surgery; 2 patients (1 complete sternectomy and 1 subtotal sternectomy) were extubated in the intensive care unit within the first 24 hours. One patient, after a total anterior chest wall demolition (total sternectomy extended to both clavicles and anterolateral ribs), needed prolonged mechanical ventilation, tracheostomy, and discharge with home ventilatory assistance for 3 months. There was no perioperative mortality. Neither major septic complications nor flap-related complications occurred: 4 patients (8.3%) developed a seroma (1 subtotal sternectomy and 3 lateral chest wall nonrigid reconstruction) and were treated conservatively without consequences. No prosthesis infection or other complications directly related to the PACLIDEM were registered. postoperative intensive care unit and hospital stay averaged 2.3 ± 9.1 and 10.8 ± 9.9 days (range, 0-63 and 4-63 days), respectively. A partial paradoxical movement occurred in 1 case (2.1%) in a patient with nonrigid reconstruction, but without respiratory complications linked to chest-wall instability. No mortality was seen through 30-days postoperatively. The mean follow-up was 33.9 ± 21.0 months and overall survival was 77%. Histologic examination showed wide resection margins in 44 patients (91.6%); 2 patients (4.2%) had marginal resection (both grade I chondrosarcoma), whereas 2 patients (4.2%) showed an intralesional R1 resection and underwent a redo surgery to enlarge the resection margins where required. We had a local recurrence in 2 patients (4.2%) 15 months and 18 months after surgery, performed for a desmoid tumor and a grade II chondrosarcoma, respectively. Both patients underwent a new radical resection. The histological examination of the surgical specimen in the patient with local recurrence from grade II chondrosarcoma includes the area where the PACLIDEM mesh was implanted at the time of first resection (18 months prior). In this specimen there was an absence of the classic foreign body reaction because it would have been observed in case of a synthetic mesh.

#### Primary benign chest wall tumors

Our series includes 4 patients with benign chest wall tumors treated with radical resection and reconstruction (2 men; median age, 42.5 years; range, 24-55 years). Histology was: aneurysmatic osseous cyst n = 3 and fibrous dysplasia n = 1.

In 2 patients (50%) a rigid reconstruction was associated with biological mesh implant. No perioperative complications were registered. At a mean follow up of 52 months morbidity was 0%.

#### Secondary chest wall tumors

Twenty-nine patients underwent surgery because of a chest wall secondary lesion (mean age, 66.8 ± 9.6 years; 19 [65.5%] men). The histologic types are depicted in Table 2. A previous chemo- and/or radiation therapy had been performed in 25 patients (86.2%). Preoperative radiation therapy on the surgical site had been performed in 7 breast cancer recurrences and 1 lymphoma.

A rigid reconstruction with titanium bars was performed in 18 (62.1%) patients, whereas a muscular flap was associated in all cases (LD n = 11, PM n = 17, transverse rectus abdominis n = 1). All patients were extubated after surgery. In 4 cases (13.8%) we had a seroma at the prosthesis and muscular-flap receiving area, always conservatively treated without infection and/or the need for invasive treatment or prosthesis removal. No case of respiratory failure was registered. Mean hospital stay was 13.5 ± 10.9 days (1.2 ± 0.9 intensive care unit stay days). The histologic examination showed wide resection margins in 27 patients (93.1%); 2 patients (6.9%) had marginal resection (both local recurrences from breast cancers). No 30-day mortality was registered. Patients had a mean follow-up of 26.7 ± 20.65 months (range, 1-71 months) and survival was 37.9%. No complications other than seromas and flap ischemia (as described above) were identified.

### Group 2: Trauma

Chest trauma was the indication for CWR with PACLIDEM in 10 (9.5%) patients of the series (6 [60%] men; mean age, 70.9 ± 14.9 years; range 40-90 years). More than 1 comorbidity was present in 8 patients (80%): diabetes 4 (40%), renal failure 1 (10%), and cerebrovascular disease 2 (20%). In all cases, chest wall stabilization and prosthetic reconstruction was necessary because of the chest wall destruction and the loss of soft tissues and bones (always >3 ribs with displaced fractures). In 3 (30%) patients, a sternal displaced fracture was associated; bone fragments were dislocated into the lung or into the mediastinum in 4 cases (40%) and a compound fracture of the clavicle was present in 2 patients (20%). The chest wall defect was always >5 cm at the major diameter and the dimension of the biological mesh used was 15 × 20 cm n = 6 (60%), 5 × 10 cm n = 3 (30%), or 20 × 30 cm n = 1 (10%). A rigid reconstruction with titanium bars was always necessary (n = 10, 100%) and muscular flap was used to cover the reconstructed area in 7 patients (70%).

We experienced 1 case (10%) of dehiscence on soft tissue at the site of the sternal wound; this case had no prosthesis infection despite the abundant foreign body materials (PACLIDEM 20 × 30 cm plus titanium bars) under the soft tissues (a bilateral PM flap) and was treated conservatively (using the vacuum assisted closure system) healing in 1 month by secondary intention. Mean hospitalization was 28.4 ± 26.8 days (range, 8-97 days) with 17.7 ± 23.8 days in the intensive care unit (range, 0-75 days). Chest and soft tissues drains were removed after 7.8 ± 4.2 and 5.9 ± 3.5 days, respectively. Overall survival at 30 days was 100%.

### Group 3: Lung Hernia

We had 12 cases (11.4% in the series; 9 men; mean age, 58.1 ± 9.9 years) of lung hernia, all but 1 following anterior minithoracotomy for mitral valve surgery (11; 91.7%); in 1 case (8.3%) lung hernia involved the anterior port after a video assisted thoracic surgery lobectomy. The chest wall defect was always repaired with a PACLIDEM 10 × 15 cm (Video 2). In all cases, the homolateral PM was mobilized to cover the defect and the prosthesis. One case (8.3%) of complication was registered, consisting of a postoperative bleeding with prosthesis displacement due to the chest wall hematoma; surgical revision was necessary with repositioning of the mesh without any other inconvenience. Mean hospitalization was 5.5 ± 2.6 days; chest and soft tissues drains were removed after 2.5 ± 1.3 and 3.9 ± 2.6 days, respectively. No 30-day morbidity and mortality were registered. At a mean follow-up of 29.1 ± 21.5 months (range, 2-67 months) we had no complications and no relapses.

### Group 4: Infectious Disease

In 2 patients (1.9%), chest wall resection and reconstruction were indicated because of an infectious disease: 1 abdominothoracic fistula due to complicated Crohn disease and 1 septic arthritis of the sternoclavicular joint with bone erosion and compression on the cervical esophagus and subclavian vessels. Both patients were treated with chest wall resection and reconstruction using PACLIDEM 10 × 15 cm. In the last case, osteomyelitis evolved and extended to the anterior chest wall after failure of conservative treatment; resection of the left sternoclavicular joint was extended also to the manubrium, first and second sternocostal cartilages, and the involved soft tissues, creating a large defect needing reconstruction. Debridement of infected tissues in this region uncovered the subclavian vessels until the origin of the innominate artery so that a mesh was placed to protect big vessels and separate them from titanium bars used to reconstruct the bones. In both cases an LD myocutaneous flap was used to cover the prosthetic materials. Hospital stays were 18 and 24 days (2 and 6 days in the intensive care unit), respectively; chest drainage lasted 2 and 6 days, whereas soft tissue drainage stayed for 17 and 9 days, respectively. Patients had no 30-day morbidity and mortality, and both are free from relapse after 17 and 15 months from surgery.

## Discussion

The goals of CWR are several: to maintain the respiratory function, to restore the chest wall rigidity avoiding its contraction, to re-establish the chest wall integrity to protect the contents of the thorax from trauma and infection, to prevent lung herniation and paradoxical chest wall motion, to ensure shoulders stability, to avoid the trapping of the scapula, and whenever possible to provide an acceptable cosmetic result. In case of extended resections and depending on the location of disease, a composite reconstruction is necessary (rigid and not-rigid materials) to replace bones, cartilage, and soft tissues. Meshes in these situations are needed to replace parietal pleura and intercostal muscles or to protect and separate the mediastinum (in the case of anterolateral resections or sternectomies) because unfortunately, rigid materials allow lung or visceral herniation between their structures. Muscle flaps are needed to replace superficial soft tissues, to separate prosthetic materials from the skin, and to put over the defect and the meshes a well vascularized tissue for a safe healing (Videos 1 and 3).

For these reasons, chest wall resection often needs a complex series of steps during reconstruction that are not always able to recreate the preoperative physiological condition. As shown in previous literature, complication rate after chest wall surgery can be very high, varying from 38% to 69%.^1^, ^2^, ^3^, ^4^^,^^11^^,^^12^ For example, in 2006, Weyant and colleagues^4^ showed that within 30 days, complication rates were 38% for rigid methylmethacrylate sandwich techniques and a 4.5% 90-day prosthesis removal rate was observed; for polytetrafluoroethylene or polypropylene mesh, the 30-day mortality was 27% and the 90-day prosthesis removal rate was 4.1%. We are far from the ideal prosthetic material, as defined in 1983 by LeRoux and Shama^18^: rigid, malleable, radiolucent, durable inexpensive, easily incorporated by the body, physically and chemically inert, resistant to infection and strain, unable to elicit inflammatory or foreign body reaction, noncarcinogenic, hypoallergenic, and sterilizable.

Although synthetic tissue materials (meshes and titanium) provide strong tissue reinforcement, they remain a source of a foreign body reaction, which can result in serious complications. This was also our historical experience, with several cases of synthetic meshes infection needing redo surgery with their removal. The introduction of biological prosthesis in thoracic surgery is a new challenge. Extracellular biological mesh provides the extracellular scaffold needed for a physiologic tissue healing; they are either derived from human (allograft; derived from dermis, intestinal mucosa, or pericardium) or animal (xenograft; usually porcine or bovine) tissues.

PACLIDEM is a collagen matrix patch derived from porcine dermis in which cells, cell debris, DNA, and RNA have been removed through a decellularization process. The resulting acellular matrix together with its constituent collagen fibers is cross-linked with hexamethylene diisocyanate to give the mesh additional stability and reduction of collagenase degradation. The biomechanical characteristics of PACLIDEM have been tested and compared with other collagen materials in experimental settings. In a porcine model of ventral incisional hernia repair, PACLIDEM demonstrated excellent biomechanical characteristics and histologic remodeling compared with other biological meshes. The tensile strengths of sites repaired with biologic mesh were not influenced by very high de novo tensile strength/stiffness or mesh-specific variables.^19^^,^^20^ Crosslinking resulted in an increased tensile strength of the tissue before a strong, mature wound has formed; this is crucial not only in abdominal hernia repair, but also in CWR,^21^ where mechanical stresses are high. These characteristics made PACLIDEM our choice for CWR.

In cases of chest wall tumors, the main objective of surgery is to achieve disease-free margins (R0).^21^ R0 can only be accomplished by an aggressive bone and soft tissues resection; depending on the extent of resection, distortion and malfunction of chest wall dynamics may ensue. Failure of the postoperative chest wall musculoskeletal system, including the area of reconstruction, to provide adequate physiological respiratory function may result in acute and potentially chronic restrictive respiratory failure. A favorable CWR method promotes early extubation and potentially reduces the risk of mortality.^4^^,^^22^^,^^23^ Respiratory failure has been reported in up to 26% of patients with large chest wall resection utilizing nonrigid reconstruction^24^^,^^25^; however, in this series an R0 resection was achieved in >90%, with only 1 case (1.2% of oncological series) of respiratory failure after surgery, with the need of prolonged postoperative mechanical ventilatory support (3 months) in a patient undergoing an extended anterior chest wall resection with total sternal titanium replacement.

Regarding infections of the sternoclavicular joint, several authors report successful treatment by debridement and closure with muscle flaps, with or without bony stabilization and no mesh.^26^ In our case, the mesh was needed to protect blood vessels from the titanium bars used to recreate chest wall stability because the chest wall resection involved the whole manubrium and a large quantity of soft tissues.

The addition of rigid prosthetic material to nonrigid biologic reconstruction systems increases the strength of the reconstruction but also raises the risk of infection.^1^, ^2^, ^3^, ^4^^,^^24^ Reconstruction using the PACLIDEM alone was successful in 48 patients, whereas in the remaining 57 the repair was achieved by adding a rigid titanium system. In 61 (58.1%) patients, a myocutaneous flap was also added. Further, anterior chest wall defects all required additional rigid fixation at least from an esthetic point of view. Despite this extensive use of prosthetic materials and myocutaneous flaps, complications related to the chest wall resection and reconstruction occurred in 14 patients (13.3%), which is acceptable.

In cases of surgical site infection, treatment should include the removal of synthetic materials. Recent evidence suggests that the resorbable features of the biological patches do not require their removal even if infected.^27^^,^^28^ In our series, we did not experience prosthesis infection, despite the surgical site being already infected (16 patients [15.2%]) or at high risk of infection (28 patients [26.7%]). None in the group of 9 patients with wound hematoma and/or seroma developed an infection. No patient needed prosthesis removal due to infection or for any reason. Reconstruction with prosthetic materials after radiotherapy also is considered a high-risk scenario; we safely used biological meshes in 2 radio-induced sarcomas among the primary tumors group and 7 breast cancer recurrences and 1 lymphoma among the secondary tumors group.

PACLIDEM withstands high tensile forces, resulting in a strong biological scaffold incorporated into the repair with the necessary properties to facilitate soft tissue healing. We therefore utilized this feature to reconstruct the chest wall in nonmalignant diseases. The device was effectively implanted on its own in 12 lung hernia patients and in 10 trauma patients without postoperative complications or further herniation. This experience in patients with benign tumor confirms previous results in repairing secondary incisional herniations.^13^^,^^14^^,^^28^^,^^29^

## Conclusions

The conclusions of this study can be summarized as follows:

• PACLIDEM represents a valuable option in CWR, especially in case of high-risk patients or infected surgical sites. PACLIDEM showed excellent results on a large series with a long follow-up.
• Excellent wound healing and long-term stability are achieved even in large defects by using biological meshes, and PACLIDEM confirms these results.
• The use of PACLIDEM was not associated with any infections; early and late postoperative complications are acceptable.

The main limits of this study are:

• There's no comparison group. A retrospective match analysis would be not feasible because the low number of cases and the need to involve a long period series, including different reconstruction techniques (with a lower technological quality of prosthetic materials).
• This is not a randomized trial, although a future randomized trial could be support based on the data from this study.

### Conflict of Interest Statement

Alessandro Gonfiotti reports receiving personal fees (consulting fees and honoraria) from Medtronic (Minneapolis, Minn), all outside the submitted work. This activity includes expert opinions on CWR techniques, lectures during surgical meetings, and training masters. All other authors reported no conflicts of interest.

The *Journal* policy requires editors and reviewers to disclose conflicts of interest and to decline handling or reviewing manuscripts for which they may have a conflict of interest. The editors and reviewers of this article have no conflicts of interest.

## Supplementary Data

Video 1 A case of subtotal sternectomy, with both clavicles resected without reconstruction. The sternal defect has been reconstructed with biological mesh, titanium, and muscle flap to cover the prosthetic material. The postoperative result was excellent from a cosmetic and functional point of view (eg, no deformities or paradoxical movement). Both arms move normally even if clavicles were not reconstructed. Video available at: https://www.jtcvs.org/article/S2666-2507(22)00100-6/fulltext.

Video 2 The chest wall defect after anterior minithoracotomy. Video available at: https://www.jtcvs.org/article/S2666-2507(22)00100-6/fulltext.

Video 3 A case of total sternectomy and reconstruction with biological mesh and 3-dimensional customized titanium implant. Video available at: https://www.jtcvs.org/article/S2666-2507(22)00100-6/fulltext.
