# Supplementary figures and images for: Chest wall reconstruction with implantable cross-linked porcine dermal collagen matrix: Evaluation of clinical outcomes
Source: JTCVS Tech. 2022 Feb 22;13:250–60. doi: 10.1016/j.xjtc.2022.01.021 (PMC9196048; doi:10.1016/j.xjtc.2022.01.021)

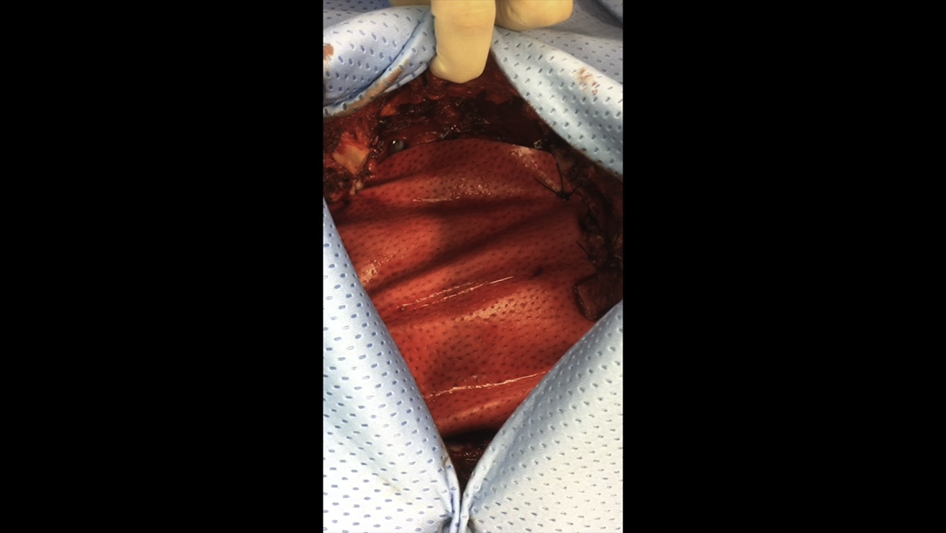

Supplement: Video 1 — A case of subtotal sternectomy, with both clavicles resected without reconstruction. The sternal defect has been reconstructed with biological mesh, titanium, and muscle flap to cover the prosthetic material. The postoperative result was excellent from a cosmetic and functional point of view (eg, no deformities or paradoxical movement). Both arms move normally even if clavicles were not reconstructed. Video available at: https://www.jtcvs.org/article/S2666-2507(22)00100-6/fulltext. [file fx2.jpg]

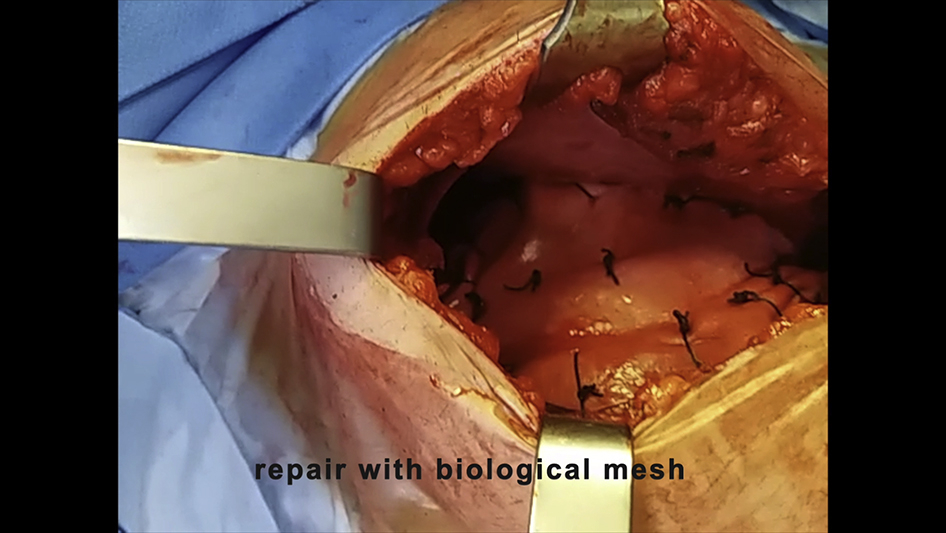

Supplement: Video 2 — The chest wall defect after anterior minithoracotomy. Video available at: https://www.jtcvs.org/article/S2666-2507(22)00100-6/fulltext. [file fx3.jpg]

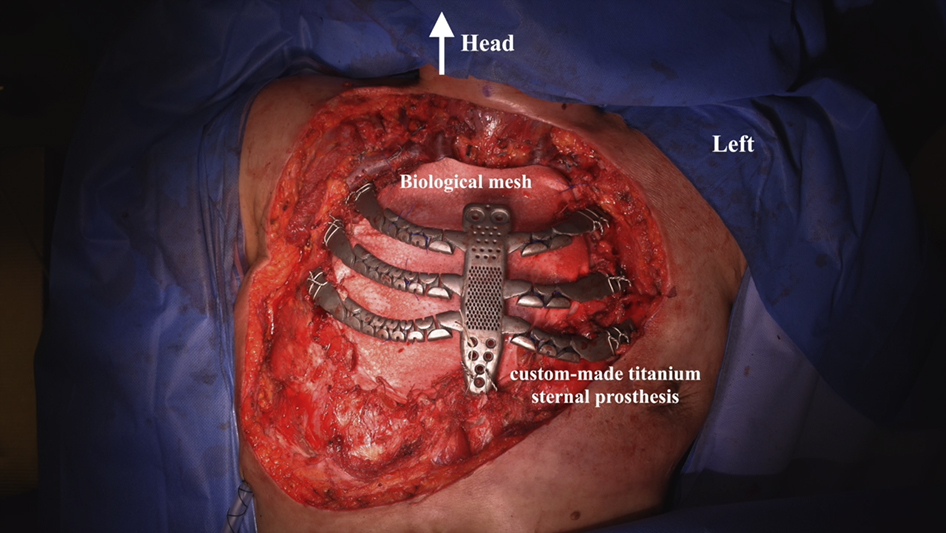

Supplement: Video 3 — A case of total sternectomy and reconstruction with biological mesh and 3-dimensional customized titanium implant. Video available at: https://www.jtcvs.org/article/S2666-2507(22)00100-6/fulltext. [file fx4.jpg]
